# Multiple Aspects of Irritable Bowel Syndrome and the Role of the Immune System: An Overview of Systematic Reviews with a Focus on Polyphenols

**DOI:** 10.3390/ijms252211993

**Published:** 2024-11-08

**Authors:** Lucia Carmela Passacatini, Sara Ilari, Saverio Nucera, Federica Scarano, Roberta Macrì, Rosamaria Caminiti, Maria Serra, Francesca Oppedisano, Jessica Maiuolo, Ernesto Palma, Valentina Malafoglia, Carlo Tomino, Massimo Fini, Vincenzo Mollace, Carolina Muscoli

**Affiliations:** 1 IRCCS San Raffaele Roma, 00166 Rome, Italy; carmela.passacatini@sanraffaele.it (L.C.P.); valentinamalafoglia@yahoo.it (V.M.); carlo.tomino@sanraffaele.it (C.T.); massimo.fini@sanraffaele.it (M.F.); 2 Department of Health Sciences, University “Magna Graecia” of Catanzaro, 88100 Catanzaro, Italy; saverio.nucera@hotmail.it (S.N.); federicascar87@gmail.com (F.S.); robertamacri85@gmail.com (R.M.); rosamariacaminiti4@gmail.com (R.C.); maria.serra@studenti.unicz.it (M.S.); foppedisano@unicz.it (F.O.); maiuolo@unicz.it (J.M.); palma@unicz.it (E.P.); mollace@unicz.it (V.M.)

**Keywords:** IBS, immune system, polyphenols

## Abstract

Irritable bowel syndrome (IBS) is a complex and often debilitating condition that significantly impacts the gastrointestinal system and the overall quality of life of those affected. IBS is characterized by a variety of distressing symptoms, including cramping, abdominal pain, and irregular bowel movements, underlined by an intricate interplay of immune system dysfunction in its pathology. Numerous studies highlight an increased cellular immune response, with elevated levels of proinflammatory cytokines, mucosal alterations due to immune imbalance, and visceral hypersensitivity. Notably, studies indicate increased levels of proinflammatory cytokines, immune imbalances that lead to mucosal changes, and heightened visceral sensitivity. The roles of effector and regulatory T cells are particularly intriguing, as their modification appears to amplify inflammation and may even contribute to autoimmune disorders. This overview of systematic reviews explores the connections between IBS and immune responses, with a focus on immune cell alterations and proliferation of lymphocytes and mast cells in affected individuals. Furthermore, we explore various aspects of IBS management, including its pharmacological approaches. A systematic search of PubMed and Web of Science yielded 676 articles, which were ultimately narrowed down to 9 key studies that met our inclusion criteria. These studies collectively underscore the activation of the immune system with the degranulation of the mast cells in patients with IBS, where the release of inflammatory mediators can compromise intestinal permeability, exacerbating symptoms further. Additionally, we examine the multifaceted management strategies for IBS, emphasizing the potential therapeutic benefits of dietary polyphenols as antioxidants. The present study aims to enhance our understanding of IBS and offer insights into more effective treatment strategies for this challenging condition.

## 1. Introduction

Irritable bowel syndrome, or IBS, is a prevalent condition affecting the digestive system. People with IBS experience a range of uncomfortable symptoms, including cramping, abdominal pain, and irregular bowel movements. These symptoms can have a significant impact on daily life. In fact, estimates suggest that IBS affects a sizeable portion of the population: 11.2% [1]. Although the etiology has not yet been well defined, the evidence is abundant on the brain–gut pathways as disease factors [2]. Women report more IBS symptoms than men, despite the diagnostic criteria employed. Globally, the overall prevalence of IBS in women is 67% higher than in men [3].

A recent interesting study highlighted an increase in gastrointestinal (GI) symptoms during menstruation and early menopause, suggesting that estrogens and progesterone may play a role in patients with IBS; also, the anatomical colon tract differences between the sexes can contribute to the differences in IBS symptoms.

Studies have reported the role of androgens in the development of chronic pain disorders and the analgesic effect of testosterone [4,5,6,7].

Immune activation may have an important pathogenic role in IBS. Many studies found an increased cellular immune response with enhanced production of proinflammatory cytokines. Patients with IBS showed higher baseline TNF-α, IL-1β, IL-6, and *Escherichia coli* lipopolysaccharide (LPS)-induced IL-6 levels than healthy controls (HCs). Analyzing IBS subgroups, all cytokine levels were significantly higher in patients with diarrhea-predominant IBS (D-IBS) [8,9]. The percentage of activated natural killer (NK) and T cells was significantly lower in the IBS group than in the control. The NK cell percentage and activity have been shown to vary in response to many psychological stressors [10,11]. Furthermore, a study revealed that a high-salt diet (HSD) triggers the modification of effector and regulatory T-cell activities and enhances tissue inflammation in autoimmune disorders, which in turn affects the prognosis. HSD induces NK-cell-mediated tumor immunity by inhibiting PD-1 expression while enhancing IFNγ and serum hippurate [12].

Gut microbiome suppression could lead to important changes in oxidative status. Recently, an overproduction of reactive oxygen species (ROS)—such as superoxide, hydroxyl radicals, hydrogen peroxide, singlet oxygen, and nitric oxide—has been observed in the gut of animals and patients with IBS. However, the precise role of these species in the pathogenesis of IBS still needs to be clarified [13]. Furthermore, the crucial role of antioxidants, particularly natural polyphenols, remains undetermined in the therapeutic management of patients with IBS [14].

### 1.1. Irritable Bowel Syndrome: An Overview

Among the gastrointestinal disorders, irritable bowel syndrome (IBS) represents the most common and debilitating one. According to the Rome criteria, IBS can be grouped in subtypes based on symptoms of constipation or diarrhea and stool consistency (IBSC, IBS-D) or a mix of the two (IBS-M, also termed IBS alternating, IBS-A). The Rome IV Diagnostic Criteria, published in May 2016, represents an unclassified (IBS-U) subgroup of understood conditions. In addition, postinfectious IBS and FD, following infectious gastroenteritis, have also been recognized as distinct subtypes (PI-IBS, PI-FD) [15].

In the absence of reliable biomarkers, diagnosis is usually based on symptom criteria.

Considering the pathogenesis of IBS, heterogeneity is a typical feature that contributes to the characteristic symptoms: cramping, abdominal pain, bloating, gas, gut microbiota alterations, activation of the gut immune system, inflammatory changes, diarrhea or constipation or both, associated with altered bowel habits in the absence of structural abnormalities [16]. IBS results as a chronic condition that reduces quality of life and work productivity, having a global prevalence of 11.2% [15]. Cognitive processes modulate pain perception: patients with IBS with chronic pain conditions have impaired attention, as well as working and episodic memory loss [17]. Many environmental factors, as well as genetic factors, distress the host microbial colonization, although infectious gastroenteritis is the strongest risk factor for the development of IBS. Studies and observations show that the intestinal microbiota can affect the digestive system and immune system responses, and there are correlations between IBS and fibromyalgia, with increased expressions of IL-1, IL-2, and TNF-α in the patients [18,19]. Patients with IBS show an emotional hyper-responsivity manifested through an increased negative affect compared to healthy controls [20,21].

The bidirectional interaction between the gut and the nervous system defines the gut–brain axis and includes the central nervous system (CNS, brain, and spinal cord), the autonomic nervous system (ANS), the enteric nervous system (ENS), and the hypothalamic pituitary adrenal (HPA) pathway [22,23]. Hippocrates, the father of modern medicine, declared in 400 B.C. that “death sits in the bowels” [24]. Psychosocial stressors can reactivate the HPA axis. Some studies demonstrated greater cortisol responsivity in patients with IBS [16]. Brain–gut interactions extend well beyond the boundaries of the stressed, anxious, or depressed gut and should include situations where the brain, gut, and their connection through the autonomic nervous system are involved. The principal subject implicated in the anxiogenic and endocrine responses to stress is the peptide corticotropin-releasing factor (CFR) [25].

A comprehensive disease model of brain–gut–microbiome interactions has emerged, which can explain the altered bowel habits, chronic abdominal pain, and psychiatric comorbidities [26].

Abnormal bowel reactivity in adults and children with functional gastrointestinal disorders (FGIDs) is frequently associated with psychological and neuropsychiatric comorbidities, and the possible relationships between FGIDs and neuropsychiatric diseases are supported by the evidence that there are several areas of abnormal brain activity in patients with FGIDs, as reported in a recent study [27].

Environmental stress, such as systemic proinflammatory cytokines, activates the secretion of CRF from the hypothalamus and stimulates adrenocorticotropic hormone (ACTH) secretion from the pituitary gland; this, in turn, leads to the release of the major stress hormone, cortisol, which affects many human organs, including the brain [28].

**Figure 1:**
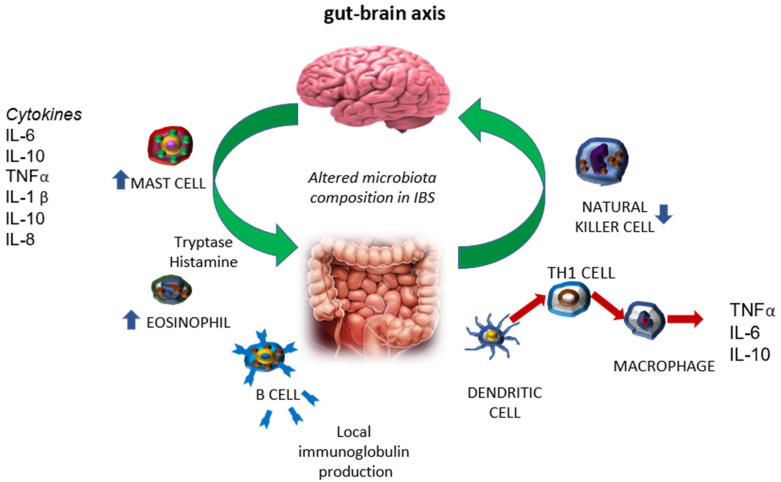
Immune factors involved in IBS.

CRF plays the role of a neuroendocrine hormone that stimulates the pituitary–adrenal (PA) axis and is a neuromodulator of behavior and autonomic nervous system (ANS) activity, which regulates visceral function under stress conditions. CRF-related peptides and CRF receptors are expressed in various sites such as the colon, endocrine system, and immune cells, where they are involved in the colonic manifestations of IBS [29]. Acute gastroenteritis following exposure to pathogens can precipitate the development of IBS, and studies have demonstrated changes in the gut microbiome in patients with IBS [30]. Lastly, serotonin (produced by the intestinal enterochromaffin cells) and histamine (produced by the mast cells in the mucosa) have been shown to affect inflammation and intestinal barrier integrity, and serotonin has been implicated in visceral hypersensitivity. The gut microbiota appears to modulate serotonin production, suggesting another potential mechanism by which gut microbes may affect the gut–brain axis and contribute to IBS symptoms. Moreover, IBS is correlated with increases in serum proinflammatory cytokines. For instance, TNF-α and IL-17 increase, while the serum levels of anti-inflammatory cytokines (i.e., IL-10) drops drastically. Moreover, clinical trials demonstrated that the plasma levels of TNFα, IL-1β, IL-6, and IL-8 were higher in patients with IBS than in controls, while the serum level of IL-10 was significantly lower (Figure 1) [31,32,33].

### 1.2. IBS and the Immune System: An Overview

The immune system is a fascinating defense system made up of a network of cells, molecules, and tissues that work together to protect the host from foreign invaders or internal abnormalities. The innate immune system includes all aspects of the host’s immune defense mechanisms, which are encoded in their mature functional forms by germ-line genes. In addition to physical barriers, such as the epithelial cell layers with tight cell–cell contacts and the secreted mucus layer that overlays the epithelium in the respiratory, gastrointestinal, and immune system, the body employs several other defenses against microbes, including in the genitourinary tract and the epithelial cilia [34]. Conventionally, in higher vertebrates, the immune system is divided into the nonspecific innate and the specific adaptive immune systems. The innate immune system represents the first line of defense in response to nonself and danger signals from microbial invasion or tissue injury. It includes cells such as basophils, dendritic cells, eosinophils, Langerhans cells, mast cells, monocytes, macrophages, neutrophils, and NK cells. The innate response also includes soluble proteins and bioactive small molecules that are either constitutively present in biological fluids, such as the complement proteins, defensins, and ficolins 1–3, or that are released from cells as they are activated [34]. The molecules released include cytokines, which regulate the function of other cells; chemokines, which attract inflammatory leukocytes; lipid mediators of inflammation; reactive free radical species; and bioactive amines and enzymes, which also contribute to tissue inflammation. Lastly, the innate immune system includes membrane-bound receptors and cytoplasmic proteins that bind the molecular patterns expressed on the surfaces of pathogens [35]. The adaptive immune system forms the second, more efficient, and specific line of defense as it retains specific memory of previously encountered antigens. There are two types of adaptive responses: the cell-mediated immune response, which is carried out by T cells; and the humoral immune response, which is mediated by activated B cells and antibodies but also depends in part on the help of T cells [32]. Activated T and B cells specific for molecular structures on the pathogen proliferate and attack the invading pathogen [33,34].

Among the immune cells, mast cell activation is the leading cause of immune activation and abdominal pain in IBS; specifically, changes in mast cell function are a hallmark of the pathophysiology of IBS, which are characterized by increased activity and the release of mediators. In addition, the degranulation of mast cells typically occurs in proximity to mucosal nerves in patients with IBS [36].

### 1.3. IBS Therapy and the Pharmaceutical Approach: Role of Polyphenols

Intestinal disorders are treated with pharmacological, psychological, and complementary methods. Pharmacological intervention relieves IBS symptoms: antispasmodic drugs (smooth muscles relaxants) such as dicyclomine and hyoscyamine are medications that ameliorate abdominal pain via relaxing the gut smooth muscle; however, they can cause constipation. The antibiotic rifaximin manages nonconstipation IBS by improving bloating and diarrhea. The anticonvulsants gabapentin and pregabalin are also applicable for chronic neuropathic pain. Pregabalin showed clinical efficacy in reducing pain in IBS and fibromyalgia [37].

Selective serotonin reuptake inhibitors, tricyclic antidepressants, 5-hydroxytryptamine type-3 antagonists such as linaclotide and ramosetron are also among the utilized medications. Moreover, probiotics play a beneficial role in intestinal function as they can protect against pathogenic bacteria via their antimicrobial properties [38].

Despite these, new therapeutic interventions are being studied due to the lack of truly effective cures for IBS [15]. In this context, the role of antioxidants such as natural polyphenols (PPs) represents a possible innovative therapeutic approach for the management of patients with IBS. Polyphenols, a large family of compounds found naturally in vegetables, have interesting anti-inflammatory and cardioprotective properties. Moreover, the interplay between polyphenols and the gut microbiota, as well as how polyphenols are metabolized to produce various polyphenolic metabolites have been demonstrated. Studies have highlighted the protective effects of polyphenols and their metabolites against various gastrointestinal disorders/diseases such as IBS [39,40]. The current therapies for the treatment and management of inflammatory gastrointestinal conditions include various dietary and lifestyle recommendations, along with herbal treatments featuring natural polyphenolic compounds. These include extracts from *Perilla frutescens*, *Aloe vera*, *Mentha piperita*, *Cynara scolymus*, *Acacia catechu*, *Camellia sinensis*, and polyphenolic compounds such as the isoflavones baicalin, anthocyanin, and quercetin [41,42,43]. Biophenols, listed among the polyphenols, are phytochemicals found in foods such as extra virgin olive oil, peanuts, turmeric, ginger, tea, and peppermint. The consumption of antioxidants has been associated with reduced levels of oxidative damage to lymphocytic DNA. Similar observations have been made with polyphenol-rich food and beverages, indicating the protective effects of polyphenols [44]. Among these phytochemicals, bergamot plays a protective role in patients with metabolic syndrome and cardiometabolic risk. New scientific evidence also supports the efficacy of natural agents in the management of the pain due to different etiologies. Polyphenols, such as bergamot, oleuropein, and resveratrol, have strong antioxidant, anti-inflammatory and analgesic properties [42,43].

Although polyphenols are the more common term for such phytochemicals, they represent only phenolic compounds with two or more aromatic benzene rings. Recently, there has been a move toward utilizing the more scientifically accurate term biophenols, which represents all plant-derived phenols. The benefits of biophenols have been suggested for the treatment of chronic conditions in humans, such as decreasing toxicity in hemodialysis, improving mental health and cognitive performance, managing nausea and vomiting in chemotherapy, or reducing cardiovascular disease risk [45,46].

The beneficial effects of biophenols are due to a variety of mechanisms, including their antioxidant, antiglycation, and anti-inflammatory activities in glucose and lipid metabolism [47,48]. Flavonoids, polyphenolic compounds of natural origin, mostly O-glycosides, are widely present in fruits and vegetables. They have multiple beneficial antiallergic, anti-inflammatory, antiproliferative, and anticarcinogenic properties. Inflammatory conditions such as inflammatory bowel disease, ulcerative colitis, and Crohn’s disease are chronic conditions, characterized by the activation of inflammatory pathways, including the nuclear factor kappa B (NFkB) and proinflammatory cytokines (e.g., TNF-a, IL-1b) pathways, and dysregulated immune responses. In fact, the inhibition of NFkB activity has been suggested to be a major component of the anti-inflammatory activity of glucocorticoids and 5-aminosalicylic acid (5-ASA), both of which are frequently used for the treatment of chronic intestinal inflammation [49]. Only few studies have been conducted on experimentally induced inflammation in in vitro and in vivo intestinal models. These models have been used to assess the beneficial health effects of dietary polyphenolic compounds (PCs) and their potential to reduce or delay IBD development in early life (Table 1).

Investigations of their mechanisms of action have shown that PCs, such as kaempferol, quercetin, apigenin, chrysin, lute olin, biochanin A, genistein, butein, paenol, ellagic acid, and resveratrol, may modulate intracellular signaling pathways via the inhibition of the activation of transcription nuclear factor B (NF B) and mitogen-activated protein kinases (MAPKs) and/or reduction of proinflammatory mediators such as interleukin (IL)-8, IL-6, and cyclooxygenase-2 (COX-2) at the mRNA and/or protein levels [60]. One of the major subclasses of flavonoids, anthocyanins, have different properties that include improving vision, cognition, and blood pressure and have protective effects against cardiovascular disease risk factors [61]. The impacts of anthocyanins on the gut microbiota include vitamin production, regulation of lipid metabolism, short-chain fatty acid production for epithelial cells, and regulation of gene expression; they also have important functional roles in the gut–brain axis relationships [61]. The anthocyanins are active in the progression of IBD, reducing the inflammation of human colon epithelial cells via the inhibition of inflammatory cytokine expression [43]. Moreover, supplementation with anthocyanins can protect against ischemia–reperfusion in the intestinal tract, an inflammatory condition that involves the high production of reactive oxygen species (ROS) and DNA damage. Numerous studies have highlighted the bioavailability of these compounds. A study reported that the microflora can improve the absorption of anthocyanins via the formation of metabolites with higher bioavailability and lead to enhanced therapeutic action in GI tissue [62]. ROS contribute to the multifaceted pathophysiology of IBS, influencing multiple aspects such as inflammation, the gut microbiota and the gut–brain axis. Oxidative stress is an essential factor leading to IBS-D, and excessive reactive nitrogen species (RNS) and ROS promote the development of IBS-D. Studies have shown that rats that are affected by IBS-D: the level of glutathione is significantly reduced and excessive amounts of ROS are produced in the intestinal cells. Excessive ROS damage cellular proteins, including cytoskeletal proteins, and eventually destroy the intestinal barrier to increase intestinal permeability. In addition, excessive ROS can induce inflammation by stimulating neutrophils, which contribute to further tissue damage [63]. During cellular transitions, intestinal epithelial cells (IECs) possess distinct metabolic identities that are reflected by changes in mitochondrial activity. The mitochondrial electron transport chain (mETC) is central to ATP production but is also a significant source of ROS, mainly superoxide [64]. Cellular metabolism, immunity, stress responses, and apoptosis are linked to mitochondrial function and to the regulation of the cellular stress response, including MT-UPR, 5′-AMP-activated protein kinase catalytic subunit α-2 (AMPK), and inflammasome activation. ROS are important mediators of mitochondrial signaling and can damage the iron–sulfur (Fe–S) center of aconitase, a mitochondrial-matrix-localized enzyme in the citrate cycle [65] (Table 2). 

The aim of this overview of systematic reviews was to explore the link between IBS and immune factors, with particular attention to the modulation of immune factors in relation to the diverse symptoms. It is well known that alterations in immune cell populations, including the increased production of lymphocytes and mast cells, contribute to the symptomatology of IBS. In this study, we examined several aspects of IBS management, including pharmacological treatment options, considering the wide range of symptoms and the absence of specific biomarkers or targeted medications.

## 2. Materials and Methods

### 2.1. Search Strategy

This overview of systematic reviews included published data from experimental studies regarding the link between IBS and the immune system, including studies in humans. For the present review, a Preferred Reporting Items for Systematic Reviews and Meta-Analyses (PRISMA)-compliant systematic search of the medical literature from the PubMed and MEDLINE databases was performed using a specific search strategy, based on a combination of words to identify relevant articles on the correlation between IBS and the immune system and, more specifically, studies on the activation of the immune system in irritable bowel syndrome. The keywords and operators used for the search of the articles were “irritable bowel syndrome and immune system; irritable bowel syndrome and activation of immunity; the role of immunity in irritable bowel syndrome”. All papers published in the period of 2019–2024 were evaluated. The reference list of all retrieved articles was also reviewed to identify other eligible studies that were not indexed by the above-mentioned databases. No additional papers were retrieved from this activity.

#### Eligibility Criteria

Screening for eligibility led to the identification of all publications that met the criteria of being original research published in peer-reviewed journals in the English language that were exclusively human-related.

Studies were excluded from the analysis for the following reasons:

- Study on IBS but not on the correlation between IBS and the immune system;
- Study on other gastrointestinal syndromes without the inclusion of IBS;
- Nonpharmacological interventions for patients with IBS except for the use of polyphenols as adjuvants;
- Animal studies;
- Cellular studies.

### 2.2. Study Outcomes

The main objective of this systematic review was to evaluate the correlation between irritable bowel syndrome and the immune system in human and animal models, considering the correlation between the typical symptoms of IBS and the activation of the immune system. The second objective was to study the different treatments for patients with IBS and, in particular, the antioxidant role of polyphenols.

## 3. Results

### 3.1. Data Collection

**Figure 2:**
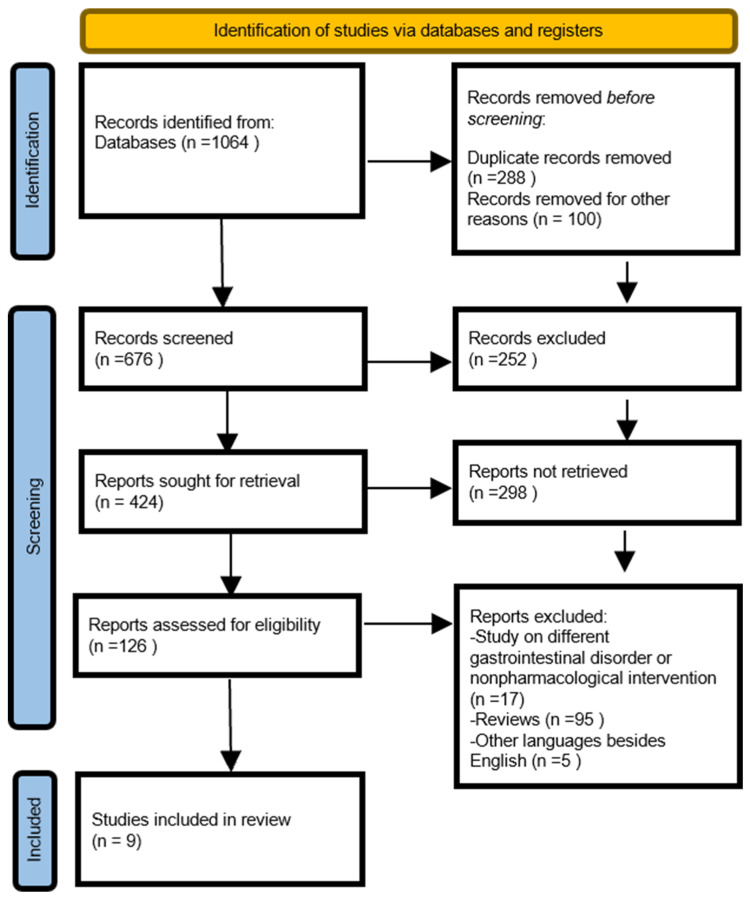
Prisma flow chart.

The systematic search identified 1064 records through a snowball search. After deduplication and title/abstract screening, the full texts of 126 articles were evaluated for eligibility; finally, 9 papers were included in the quantitative analysis. Figure 2 (PRISMA flow chart) illustrates the literature screening process. The nine studies included a total number of 2.399 patients with IBS, primarily adults. Only one study looked at 864 pediatric patients exclusively (patients were grouped for the cell counts of different gut traits).

### 3.2. Overview of Systematic Reviews on the Link Between Irritable Bowel Syndrome and the Immune System

The nine papers selected focused on irritable bowel syndrome and its correlation with the immune system. The principal and more common treatments for IBS are summarized in Table 1. Evident interactions between the immune system and stress response have been reported in various studies as mechanisms in IBS outbreaks, with low-grade inflammation or immune activation (increases in the number of mast cells and lymphocytes, cytokine-related alterations) [36]. Moreover, certain types of stressors that combine emotional and fear-eliciting features might have a greater impact on patients with IBS.

Wouters et al. reported that chronic stress alters the T helper 1/T helper 2 (Th1/Th2) pathways, with the production of myeloid-derived suppressor cells in the peripheral blood and bone marrow being associated with immunosuppression. In addition, the IL-10/STAT3 axis appears to be activated, resulting in the suppression of the production of the proinflammatory cytokine IL-12 [68,69].

Some studies have evaluated colonic motor activity using the motility index and its correlation with stress conditions in IBS. 

There is significantly increased colonic motility in patients with IBS compared to healthy controls [70]. Psychosocial stressors effectively induce systemic humoral and cellular immune responses, with an increase in circulating leukocytes [70]. Schaper conducted a systematic study on the influence of the emotional responses to acute psychological stressors using self-report questionnaires, the state version of the State-Trait Anxiety Inventory (STAI-S). Stress was predominantly evaluated using a visual analogue scale (VAS). A complete list of the mainly unpleasant emotions measured by various standardized and nonstandardized assessment tools was presented. Studies in patients with IBS have shown increased emotional hyperresponsivity compared to healthy controls, manifesting as a heightened negative affect, including higher levels of subjective anxiety, stress, anger, and hostility [16]. The intestinal mucosa is a key factor in humans with IBS, and, in general, the intricate enteric immune system comprises a large diversity of immune cells. Krammer et al [71] analyzed some studies on mast cell (MC) numbers or density in patients with IBS compared to healthy controls. They were significantly increased in most of the studies. No significant difference in the MC number was found between patients with IBS and controls in four studies. An increased number of mast cells (MCs) was most commonly observed in individuals with diarrhea-predominant IBS (IBS-D), followed by those with constipation-predominant IBS (IBS-C). The study focusing on the correlation between IBS symptoms and MC number/or density used a subset of different questionnaires [70]. Several studies have focused on the correlation between processes including chronic pain, stress response, and immune activation. Wong et al. investigated the relationship between pain and cognitive dysfunctions: patients with chronic pain conditions were found to have impaired attention, working memory, episodic memory, and executive functioning [17]. In a meta-analysis of neuroimaging studies of neural network activities in rectal distension, salience overactivity (anterior insula and anterior middle cingulate cortex), emotional arousal (pregenual anterior cingulate cortex, amygdala, hippocampus), and autonomic networks (subgenual anterior cingulate cortex) were found to be underactive. However, patients with IBS were found to have reduced dorsolateral prefrontal cortex/pre-supplementary motor area connectivity, implicating impairment in cognitive flexibility [17]. Moreover, Krammer et al. [71] focused on the effect of epigenetic mechanisms, environmental factors, and behavioral changes on gene expression, intestinal epithelial barrier dysfunction, and increased immune activation. Understanding how epigenetic regulatory pathways function might particularly aid in the identification of biomarkers for the diagnosis and treatment of cancer, autoimmune, inflammatory, and rare diseases [70]. The intricate enteric immune system comprises a large variety of immune cells. In patients with IBS, an increased occurrence of immune cells in the lamina propria compared to healthy controls has been reported. Again, an increased number of MCs has been documented most frequently in diarrhea-predominant IBS (IBS-D), followed by in constipation-predominant IBS (IBS-C) [72,73]. Studies show that patients with IBS are genetically susceptible to inflammation, with changes in pro- and anti-inflammatory cytokine types as well as an increased number of mast cells in their colon. Mast cells were increased in the ileum but not in the duodenum or jejunum of patients with IBS [74]. The contribution of mast cells to visceral pain has been suggested because of the increased mast cell infiltration in the lamina propria of the colon, ileum, and jejunum and because activated mast cells in close proximity to colonic nerves correlate with the severity and frequency of abdominal pain perception [73]. Among the immune cells, eosinophil cells, first discovered by Paul Ehrlich in 1879, are granulocytes derived from pluripotent myeloid progenitor cells. Zoltán Kiss reported considerable elevations in cell numbers and activity in several pathologic conditions affecting the GI tract in children. In patients with functional abdominal pain (defined by the Rome IV criteria) and macroscopically normal mucosa (e.g., IBS), histology could reveal an increased number of tissue eosinophils [75]. Eosinophils are multifunctional granulocytes able to release cytotoxic proteins, regulatory cytokines, and chemokines. Eosinophils reside in the gut, exerting homeostatic functions, including maintaining the protective integrity of the mucosal barrier and contributing to gut-associated immunity. In fact, duodenal immune activation was also linked to impaired mucosal integrity as well as neuronal and structural alterations in patients with functional dyspepsia [76,77]. The protective role of the mucus is further enhanced by the presence of antibacterial substances, such as the α-defensins and lysozymes secreted by the Paneth cells of small intestinal crypts and the IgA produced by the plasma cells of the lamina propria. It has been reported that IL-6 levels are increased in the blood samples from patients with IBS compared to controls, and levels of TNF-α and circulating IL-8 were increased [15]. Studies have examined the sigmoid colon, ascending colon, rectal biopsies and the descending colon. IL-10, IFN-γ, and IL-1β have been investigated in the colon, but the results remain conflicting [15] (Table 3).

Wang et al. [79] reported the role of ketotifen in a prospective, randomized, placebo-controlled study. Ketotifen is a noncompetitive histamine antagonist and mast cell stabilizer that has been shown to inhibit the release of allergy mediators by sensitized mast cells. This can stabilize the mast cell membrane and prevent cell membrane lysis and degranulation, reducing the release of various inflammatory mediators, including tryptase and histamine. The ketotifen patients group had fewer mast cells in the terminal ileum compared to the control group (Table 3) [80,81]. Moreover, the immune and inflammatory pathways are influenced by the microbiome. QX NG et al. [82] showed the important connection between the microbiome and IBS using multiomics studies, such as genomics, transcriptomics, proteomics, and metabolomics. Immune anti-inflammation-related pathways were enriched among patients with IBS-D and IBS-C compared to healthy samples [83,84].

These multiomics studies have important implications in the study of diet in patients with IBS. One study on epigenetic changes identified purine metabolism as a novel host–microbial metabolic pathway in patients with IBS-D and IBS-C [82].

## 4. Discussion and Conclusions

The present overview of systematic reviews provided a comprehensive analysis of the studies on the complex interplay between irritable bowel syndrome (IBS) and immune system activation over the last 6 years. IBS is characterized by chronic abdominal pain or discomfort, stool irregularity, and bloating, with treatment approaches including both pharmacologic and psychological strategies. However, the pathobiological mechanisms underlying IBS remain insufficiently understood, often resulting in modest treatment efficacy.

The therapies for IBS have proven to be only modestly efficacious, likely because of the limited understanding of the pathobiological mechanisms underlying this disorder. Since the pharmacological management and the rehabilitation result often vary, a dietary intervention integrating polyphenols appears to be valid. Lambarth [78] examined the literature on the role of oral and parenteral antineuropathic agents in the management of pain in patients with IBS. Given the potential for neuropathic mechanisms to drive symptoms in IBS and for some antineuropathic medicines to alleviate pain in IBS and other nociplastic conditions, there is a rationale for considering antineuropathic agents as part of the IBS treatment regime. Several studies have confirmed that neuropathic pain produces thermal hyperalgesia and allodynia, contributing to the development of reactive oxygen and nitrogen species (ROS, RNS) and cytokines, which are able to exert the oxidative, nitrosative, and nitrative effects implicated in states of persistent pain [78].

Numerous are the interactions reported between the immune system and the stress response as a mechanism of IBS, along with low-grade inflammation or immune activation. Psychosocial stressors effectively induced systemic humoral and cellular immune responses, with an increase in circulating leukocytes [16]. The correlation between mast cell activity and visceral pain emphasizes the role of these immune cells in mediating IBS symptoms, suggesting that immune activation is a fundamental aspect of IBS pathology [56]. Furthermore, the influence of psychosocial stressors on IBS is substantial, with evidence demonstrating that emotional stress significantly impacts motility and immune responses. Additionally, the interplay between pain, cognitive dysfunction, and IBS complicates the syndrome, creating a cycle of increased pain perception and impaired cognitive function [17].

Here, we showed that the intestinal mucosa plays a crucial role in IBS, with research by Krammer [71] indicating a significant increase in mast cell density, particularly in patients with diarrhea-predominant IBS (IBS-D). While some studies found no significant differences in mast cell numbers, there is a general consensus on the increased presence of mast cells in patients with IBS, especially in the lamina propria of the colon.

Chronic pain conditions, linked with the stress response and immune activation, have been shown to correlate with cognitive dysfunctions in patients with IBS, impacting attention, working memory, and executive functioning. Wong et al. noted that the interplay between chronic pain and cognitive impairment complicates IBS symptomatology [13].

Research also emphasizes the role of eosinophils, which contribute to maintaining gut integrity and immunity. An increase in these cells has been documented in IBS, with evidence suggesting their involvement in the inflammatory response and visceral pain. Furthermore, elevated levels of inflammatory cytokines, such as IL-6 and TNF-α, have been reported in patients with IBS [80].

The gut microbiome’s influence on IBS has become a focal point of research, indicating that dysbiosis can affect immune activation and contribute to IBS symptoms. Multiomics studies highlighted the potential of dietary interventions and emphasized the need for personalized approaches in IBS management, considering both genetic predispositions and lifestyle factors [81].

In conclusion, systematic reviews have elucidated the intricate interplay between the immune system and IBS. The evidence supports a model where immune activation, psychosocial stressors, cognitive factors, and microbiome alterations converge to influence the pathophysiology of IBS. Future research should focus on longitudinal studies to better understand these interactions and their implications for treatment. Investigating targeted therapies that address both immune dysfunction and psychosocial factors may lead to more effective management strategies for patients with IBS. Understanding the underlying mechanisms will be crucial for developing biomarkers that can aid in the diagnosis and personalized treatment of this multifaceted syndrome [85,86].

### Limitations of This Study

The strengths and possible methodological limitations of this review merit careful consideration: most of the studies focused mainly on the activation of mast cells and lymphocytes; therefore, there was no comparison with other immune cells such as Natural killer cells, especially in the last 5 years. Furthermore, the comprehensive data from the articles could be more precise and were sometimes conflicting, given the variegate symptoms and difficulties in treatment.

## Figures and Tables

**Table 1:** A summary of the effects of polyphenols on IBS.

Polyphenols	Source	Effect on IBS	References
Oleuropein	Olive oil (*Olea europaea* L.)	Metabolic and vascular protective effects, amelioration of gut microbiota dysbiosis, and immunomodulatory properties	[50]
Resveratrol	Grapes, strawberry, raspberry, blueberry, cocoa	Reducing of depression- and anxiety-like behaviors and intestinal dysfunction in mice	[51,52,53]
Aloe barbadensis Mill Extract(AVH200^®^)	Aloe barbadensis Mill	Higher reduction in symptom severity in treatment group	[54]
Curcumin	Rhizome of turmeric (*Curcuma longa*)	Reduction in abdominal pain and bloating in patients with IBS	[55,56]
Flavonoids (Catechins)	Green tea leaves (*Camellia* *sinensis*)	Improved host health status via modulating signaling transduction, regulation of the gut–brain axis, and maintaining mucus/intestinal barrier integrity in the gut	[57,58]
Quercetin	Black berry, apple, onion	Attenuation of visceral hypersensitivity and 5-hydroxytryptamine availability in post inflammatory irritable bowel syndrome in rats: visceral pain threshold of PI-IBS rats was markedly decreased	[59]

**Table 2:** Drugs used to treat IBS symptoms.

Drugs	Class of Drugs	Effect	Side Effect	References
Dicyclomine and hyoscamine	Antispasmodic drugs	relaxing the gut smooth muscle	Constipation	[66]
Rifaximin	Antibiotic	Improving bloating and diarrhea	Abdominal pain, nausea, vomiting, headache	[49,60]
Gabapentin and pregabalin	Anticonvulsants	Abdominal pain, urgency, and bloating	Dizziness and drowsiness	[36,61]
Tricyclic antidepressants (TCAs)	Antidepressant	Improve global symptoms of IBS, and reduce pain perception and discomfort	Hypotension, drowsiness, and constipation	[67]
Dextofisopam, benzodiazepines	Antidepressants	Antinociceptive benefits	Abdominal pain, influenza, and nausea	[63,64]
Clonidine	Alpha-2 adrenergic agonist	Attenuating fast colonic tone, reducing postprandial gastric volume, alleviating abdominal pain sensation, and enhancing colonic compliance	Drowsiness, dry mouth, and sleep problems	[65,66,67]

**Table 3:** IBS and the immune system.

Syndrome	Analyzed Cases	Immune System	Immune Alteration Site	References
IBS with increased colonic motility	37	Increase in circulating leukocytes, mast cells, T cells, macrophages: IBS < HC	Rectum, colon	[16]
IBS	13	Increased immune activation	\	[17]
IBS	36	Increased immune activation	Lamina propria, colon	[78]
Diarrhea-predominant IBS and constipation-predominant IBS	12	Increased number of mast cells	Duodenum, jejunum, and ileum	[72]
IBS	11	Increased number of mast cells and proinflammatory and immunoregulatory cytokines (e.g., IL-1,IL-3, IL-4, IL-5, IL-6, IL-10, IL-13, IL-16, tumor necrosis factor-α and transforming growth factor-β as well as chemokines (e.g., monocyte chemoattractant protein)	Colon along the small intestine from the jejunum to the ileo-cecal region	[73]
IBS and abdominal pain	52	Increased number of tissue eosinophils	Duodenum, terminal ileum	[74]
IBS	51	Increased T cells, IgG, IL-6, TNF-α, IL-8	Lamina propria cell circulation	[15]
Patients with IBS after treatment with ketotifen	19	Decreased number of mast cells in the terminal ileum and decreased percentages of degranulated mast cells in the sigmoid colon, ascending colon, and terminal ileum after treatment compared with before treatment	Sigmoid colon, ascending colon, and terminal ileum	[75]
Patients with IBS	16	Alterations in host mucosal immune response to microbial pathogens compared to healthy controls, patients with IBS had significantly increased expression of DKFZP564O0823 (an uncharacterized gene); immune- and inflammation-related pathways were enriched among patients with IBS-D and IBS-C compared to healthy subjects; increased purine breakdown by gut microbiota in patients with IBS; *Halobiforma nitratireducens*, an Archaea, was consistently elevated in flare samples from patients with IBS-D and IBS-C	Colon biopsy samples, mucosal biopsy and stool samples	[71]

## References

[1] Grayson M. (2016). Irritable bowel syndrome. Nature.

[2] Fond G., Loundou A., Hamdani N., Boukouaci W., Dargel A., Oliveira J., Roger M., Tamouza R., Leboyer M., Boyer L. (2014). Anxiety and depression comorbidities in irritable bowel syndrome (IBS): A systematic review and meta-analysis. Eur. Arch. Psychiatry Clin. Neurosci..

[3] Card T., Canavan C., West J. (2014). The epidemiology of irritable bowel syndrome. CLEP.

[4] Alvisi S., Ceccarani C., Foschi C., Baldassarre M., Lami A., Severgnini M., Camboni T., Consolandi C., Seracchioli R., Meriggiola M.C. (2023). Effect of ospemifene on vaginal microbiome in postmenopausal women with vulvovaginal atrophy. Menopause.

[5] Namazi M., Moghadam Z.B., Zareiyan A., Jafarabadi M. (2020). Exploring the impact of endometriosis on women’s lives: A qualitative study in Iran. Nurs..

[6] Kim Y.S., Kim N. (2018). Sex-Gender Differences in Irritable Bowel Syndrome. J. Neurogastroenterol. Motil..

[7] Sun Q.H., Liu Z.J., Zhang L., Wei H., Song L.J., Zhu S.W., He M.B., Duan L.P. (2021). Sex-based differences in fecal short-chain fatty acid and gut microbiota in irritable bowel syndrome patients. J. Dig. Dis..

[8] Bisping G., Lügering N., Lütke-Brintrup S., Pauels H.G., Schürmann G., Domschke W., Kucharzik T. (2001). Patients with inflammatory bowel disease (IBD) reveal increased induction capacity of intracellular interferon-gamma (IFN-gamma) in peripheral CD8+ lymphocytes co-cultured with intestinal epithelial cells. Clin. Exp. Immunol..

[9] Liebregts T., Adam B., Bredack C., Gururatsakul M., Pilkington K.R., Brierley S.M., Blackshaw A.L., Gerken G., Talley N.J., Holtmann G. (2011). Small Bowel Homing T Cells Are Associated With Symptoms and Delayed Gastric Emptying in Functional Dyspepsia. Am. J. Gastroenterol..

[10] Motzer S.A., Jarrett M., Heitkemper M.M., Tsuji J. (2002). Natural Killer Cell Function and Psychological Distress in Women with and without Irritable Bowel Syndrome. Biol. Res. For. Nurs..

[11] Cristiani C.M., Garofalo C., Passacatini L.C., Carbone E. (2020). New avenues for melanoma immunotherapy: Natural Killer cells?. Scand. J. Immunol..

[12] Rizvi Z.A., Dalal R., Sadhu S., Kumar Y., Kumar S., Gupta S.K., Tripathy M.R., Rathore D.K., Awasthi A. (2021). High-salt diet mediates interplay between NK cells and gut microbiota to induce potent tumor immunity. Sci. Adv..

[13] Balmus I., Ciobica A., Trifan A., Stanciu C. (2016). The implications of oxidative stress and antioxidant therapies in Inflammatory Bowel Disease: Clinical aspects and animal models. Saudi J. Gastroenterol..

[14] Vannucchi M., Evangelista S. (2018). Experimental Models of Irritable Bowel Syndrome and the Role of the Enteric Neurotransmission. JCM.

[15] Burns G., Carroll G., Mathe A., Horvat J., Foster P., Walker M.M., Talley N.J., Keely S. (2019). Evidence for Local and Systemic Immune Activation in Functional Dyspepsia and the Irritable Bowel Syndrome: A Systematic Review. Am. J. Gastroenterol..

[16] Schaper S.J., Stengel A. (2022). Emotional stress responsivity of patients with IBS–a systematic review. J. Psychosom. Res..

[17] Wong K.M., Yuen S.S., Mak A.D. (2019). Neurocognitive Characteristics of Individuals with Irritable Bowel Syndrome. East. Asian Arch. Psychiatry.

[18] Garofalo C., Cristiani C.M., Ilari S., Passacatini L.C., Malafoglia V., Viglietto G., Maiuolo J., Oppedisano F., Palma E., Tomino C. (2023). Fibromyalgia and Irritable Bowel Syndrome Interaction: A Possible Role for Gut Microbiota and Gut-Brain Axis. Biomedicines.

[19] Elsenbruch S., Lovallo W.R., Orr W.C. (2001). Psychological and Physiological Responses to Postprandial Mental Stress in Women With the Irritable Bowel Syndrome. Psychosom. Med..

[20] Barrio C., Arias-Sánchez S., Martín-Monzón I. (2022). The gut microbiota-brain axis, psychobiotics and its influence on brain and behaviour: A systematic review. Psychoneuroendocrinology.

[21] Morais L.H., Schreiber IV H.L., Mazmanian S.K. (2021). The gut microbiota–brain axis in behaviour and brain disorders. Nat. Rev. Microbiol..

[22] Maiuolo J., Gliozzi M., Musolino V., Carresi C., Scarano F., Nucera S., Scicchitano M., Oppedisano F., Bosco F., Ruga S. (2021). The Contribution of Gut Microbiota–Brain Axis in the Development of Brain Disorders. Front. Neurosci..

[23] Maiuolo J., Musolino V., Gliozzi M., Carresi C., Scarano F., Nucera S., Scicchitano M., Oppedisano F., Bosco F., Macri R. (2022). Involvement of the Intestinal Microbiota in the Appearance of Multiple Sclerosis: Aloe vera and Citrus bergamia as Potential Candidates for Intestinal Health. Nutrients.

[24] Kleisiaris C.F., Sfakianakis C., Papathanasiou I.V. (2014). Health care practices in ancient Greece: The Hippocratic ideal. J. Med. Ethics Hist. Med..

[25] Dunn A.J., Berridge C.W. (1990). Physiological and behavioral responses to corticotropin-releasing factor administration: Is CRF a mediator of anxiety or stress responses?. Brain Res. Brain Res. Rev..

[26] Dong T.S., Mayer E. (2024). Advances in Brain-Gut-Microbiome Interactions: A Comprehensive Update on Signaling Mechanisms, Disorders, and Therapeutic Implications. Cell Mol. Gastroenterol. Hepatol..

[27] Zhong B., Liu Q. (2021). Medical Insights from Posts About Irritable Bowel Syndrome by Adolescent Patients and Their Parents: Topic Modeling and Social Network Analysis. J. Med. Internet Res..

[28] Kazunori K., Yasumasa I., Makoto D. (2021). Hypothalamic Regulation of Corticotropin-Releasing Factor under Stress and Stress Resilience. Int. J. Mol. Sci..

[29] Stengel A., Taché Y. (2014). CRF and urocortin peptides as modulators of energy balance and feeding behavior during stress. Front. Neurosci..

[30] Pimentel M., Lembo A. (2020). Microbiome and Its Role in Irritable Bowel Syndrome. Dig. Dis. Sci..

[31] Bennet S.M.P., Palsson O., Whitehead W.E., Barrow D.A., Törnblom H., Öhman L., Simrén M., van Tilburg M.A.L. (2018). Systemic cytokines are elevated in a subset of patients with irritable bowel syndrome but largely unrelated to symptom characteristics. Neurogastroenterol. Motil..

[32] Choghakhori R., Abbasnezhad A., Hasanvand A., Amani R. (2017). Inflammatory cytokines and oxidative stress biomarkers in irritable bowel syndrome: Association with digestive symptoms and quality of life. Cytokine.

[33] Liebregts T., Adam B., Bredack C., Röth A., Heinzel S., Lester S., Downie-Doyle S., Smith E., Drew P., Talley N.J. (2007). Immune activation in patients with irritable bowel syndrome. Gastroenterology.

[34] Hillion S., Arleevskaya M.I., Blanco P., Bordron A., Brooks W.H., Cesbron J.Y., Kaveri S., Vivier E., Renaudineau Y. (2020). The Innate Part of the Adaptive Immune System. Clinic Rev. Allerg. Immunol..

[35] Chaplin D.D. (2010). Overview of the immune response. J. Allergy Clin. Immunol..

[36] Remoortel S.V., Hussein H., Boeckxstaens G. (2024). Mast cell modulation: A novel therapeutic strategy for abdominal pain in irritable bowel syndrome. Cell Rep. Med..

[37] Ilari S., Passacatini L.C., Malafoglia V., Oppedisano F., Maiuolo J., Gliozzi M., Palma E., Tomino C., Fini M., Raffaeli W. (2022). Tantali fibromyalgic supplicium: Is there any relief with the antidepressant employment? A systematic review. Pharmacol. Res..

[38] Roudsari N.M., Lashgari N.-A., Momtaz S., Farzaei M.H., Marques A.M., Abdolghaffari A.H. (2019). Natural polyphenols for the prevention of irritable bowel syndrome: Molecular mechanisms and targets; a comprehensive review. DARU J. Pharm. Sci..

[39] Plamada D., Vodnar D.C. (2021). Polyphenols—Gut Microbiota Interrelationship: A Transition to a New Generation of Prebiotics. Nutrients.

[40] Lauro F., Ilari S., Giancotti L.A., Ventura C.A., Morabito C., Gliozzi M., Malafoglia V., Palma E., Paolino D., Mollace V. (2016). Pharmacological effect of a new idebenone formulation in a model of carrageenan-induced inflammatory pain. Pharmacol. Res..

[41] Lauro F., Giancotti L.A., Ilari S., Dagostino C., Gliozzi M., Morabito C., Malafoglia V., Raffaeli W., Muraca M., Goffredo B.M. (2016). Inhibition of Spinal Oxidative Stress by Bergamot Polyphenolic Fraction Attenuates the Development of Morphine Induced Tolerance and Hyperalgesia in Mice. PLoS ONE.

[42] Muscoli C., Lauro F., Dagostino C., Ilari S., Giancotti L.A., Gliozzi M., Costa N., Carresi C., Musolino V., Casale F. (2014). Olea Europea-derived phenolic products attenuate antinociceptive morphine tolerance: An innovative strategic approach to treat cancer pain. J. Biol. Regul. Homeost. Agents.

[43] Ilari S., Lauro F., Giancotti L.A., Malafoglia V., Dagostino C., Gliozzi M., Condemi A., Maiuolo J., Oppedisano F., Palma E. (2021). The Protective Effect of Bergamot Polyphenolic Fraction (BPF) on Chemotherapy-Induced Neuropathic Pain. Pharmaceuticals.

[44] Durazzo A., Lucarini M., Souto E.B., Cicala C., Caiazzo E., Izzo A.A., Novellino E., Santini A. (2019). Polyphenols: A concise overview on the chemistry, occurrence, and human health. Phytother. Res..

[45] Agnieszka M., Jurek J., Owczarek M., Guerrera I., Torrisi S.A., Castellano S., Grosso G., Alshatwi A., Godos J. (2023). Polyphenol-Rich Beverages and Mental Health Outcomes. Antioxidants.

[46] Lin K., Li Y., Toit E.D., Wendt L., Sun J. (2021). Effects of Polyphenol Supplementations on Improving Depression, Anxiety, and Quality of Life in Patients With Depression. Front. Psychiatry.

[47] Igwe E.O., Charlton K.E., Probst Y.C., Kent K., Netzel M.E. (2019). A systematic literature review of the effect of anthocyanins on gut microbiota populations. J. Human. Nutr. Diet..

[48] Maiuolo J., Gliozzi M., Carresi C., Musolino V., Oppedisano F., Scarano F., Nucera S., Scicchitano M., Bosco F., Macri R. (2021). Nutraceuticals and Cancer: Potential for Natural Polyphenols. Nutrients.

[49] Laurindo L.F., Santos A.R.D.O.D., Carvalho A.C.A.D., Bechara M.D., Guiguer E.L., Goulart R.D.A., Vargas Sinatora R., Araújo A.C., Barbalho S.M. (2023). Phytochemicals and Regulation of NF-kB in Inflammatory Bowel Diseases: An Overview of In Vitro and In Vivo Effects. Metabolites.

[50] Chiu H.F., Venkatakrishnan K., Golovinskaia O., Wang C.K. (2021). Gastroprotective Effects of Polyphenols against Various Gastro-Intestinal Disorders: A Mini-Review with Special Focus on Clinical Evidence. Molecules.

[51] Xu Y., Cui S.Y., Ma Q., Shi J., Yu Y., Li J.X., Zheng L., Zhang Y., Si J.M., Yu Y.C. (2018). trans-Resveratrol Ameliorates Stress-Induced Irritable Bowel Syndrome-Like Behaviors by Regulation of Brain-Gut Axis. Front. Pharmacol..

[52] Zorzi M., Gai F., Medana C., Aigotti R., Morello S., Peiretti P.G. (2020). Bioactive Compounds and Antioxidant Capacity of Small Berries. Foods.

[53] Shi Y., Zhou J., Jiang B., Miao M. (2017). Resveratrol and inflammatory bowel disease. Ann. N. Y. Acad. Sci..

[54] Størsrud S., Pontén I., Simrén M. (2015). A Pilot Study of the Effect of Aloe barbadensis Mill. Extract (AVH200®) in Patients with Irritable Bowel Syndrome: A Randomized, Double-Blind, Placebo-Controlled Study. J. Gastrointestin Liver Dis..

[55] Chiarioni G., Popa S.L., Ismaiel A., Pop C., Dumitrascu D.I., Brata V.D., Duse T.A., Incze V., Surdea-Blaga T. (2023). The Effect of Polyphenols, Minerals, Fibers, and Fruits on Irritable Bowel Syndrome: A Systematic Review. Nutrients.

[56] Ng Q.X., Soh A.Y.S., Loke W., Venkatanarayanan N., Lim D.Y., Yeo W.S. (2018). A Meta-Analysis of the Clinical Use of Curcumin for Irritable Bowel Syndrome (IBS). J. Clin. Med..

[57] Pérez-Burillo S., Navajas-Porras B., López-Maldonado A., Hinojosa-Nogueira D., Pastoriza S., Rufián-Henares J.Á. (2021). Green Tea and Its Relation to Human Gut Microbiome. Molecules.

[58] Afzal M., Safer A.M., Menon M. (2015). Green tea polyphenols and their potential role in health and disease. Inflammopharmacology.

[59] Qin H.Y., Zang K.H., Zuo X., Wu X.A., Bian Z.X. (2019). Quercetin Attenuates Visceral Hypersensitivity and 5-Hydroxytryptamine Availability in Postinflammatory Irritable Bowel Syndrome Rats: Role of Enterochromaffin Cells in the Colon. J. Med. Food..

[60] Sergent T., Piront N., Meurice J., Toussaint O., Schneider Y.-J. (2010). Anti-inflammatory effects of dietary phenolic compounds in an in vitro model of inflamed human intestinal epithelium. Chem. Biol. Interact..

[61] Qin H.-Y. (2014). Impact of psychological stress on irritable bowel syndrome. WJG.

[62] Sodagari H.R., Farzaei M.H., Bahramsoltani R., Abdolghaffari A.H., Mahmoudi M., Rezaei N. (2015). Dietary anthocyanins as a complementary medicinal approach for management of inflammatory bowel disease. Expert. Rev. Gastroenterol. Hepatol..

[63] Zhang H.-Y., Zhang Y., Zhang Y., Jiang Z.-P., Cui Y.-L., Wang Q.-S. (2022). ROS-responsive thioketal-linked alginate/chitosan carriers for irritable bowel syndrome with diarrhea therapy. Int. J. Biol. Macromol..

[64] Tjong Y.-W., Ip S.-P., Lao L., Wu J., Fong H.H.S., Sung J.J.Y., Berman B., Che C.-T. (2011). Role of neuronal nitric oxide synthase in colonic distension-induced hyperalgesia in distal colon of neonatal maternal separated male rats: Elevated nNOS expression from distal colon of NMS rats. Neurogastroenterol. Motil..

[65] Rath E., Moschetta A., Haller D. (2018). Mitochondrial function—gatekeeper of intestinal epithelial cell homeostasis. Nat. Rev. Gastroenterol. Hepatol..

[66] Colomier E., Algera J., Melchior C. (2021). Pharmacological Therapies and Their Clinical Targets in Irritable Bowel Syndrome With Diarrhea. Front. Pharmacol..

[67] Qin D., Yue L., Xue B., Chen M., Tang T.-C., Zheng H. (2019). Pharmacological treatments for patients with irritable bowel syndrome: An umbrella review of systematic reviews and meta-analyses. Medicine.

[68] De Filippis F., Pellegrini N., Vannini L., Jeffery I.B., La Storia A., Laghi L., Serrazanetti D.I., Di Cagno R., Ferrocino I., Lazzi C. (2016). High-level adherence to a Mediterranean diet beneficially impacts the gut microbiota and associated metabolome. Gut.

[69] Wouters M.M., Van Wanrooy S., Nguyen A., Dooley J., Aguilera-Lizarraga J., Van Brabant W., Garcia-Perez J.E., Van Oudenhove L., Van Ranst M., Verhaegen J. (2016). Psychological comorbidity increases the risk for postinfectious IBS partly by enhanced susceptibility to develop infectious gastroenteritis. Gut.

[70] Lee K.J., Kim J.H., Cho S.W. (2005). Gabapentin reduces rectal mechanosensitivity and increases rectal compliance in patients with diarrhoea-predominant irritable bowel syndrome. Aliment. Pharmacol. Ther..

[71] Krammer L., Sowa A.S., Lorentz A. (2019). Mast Cells in Irritable Bowel Syndrome: A Systematic Review. JGLD.

[72] Rahimi R., Nikfar S., Rezaie A., Abdollahi M. (2009). Efficacy of tricyclic antidepressants in irritable bowel syndrome: A meta-analysis. WJG.

[73] Xie C., Tang Y., Wang Y., Yu T., Wang Y., Jiang L., Lin L. (2015). Efficacy and Safety of Antidepressants for the Treatment of Irritable Bowel Syndrome: A Meta-Analysis. PLoS ONE.

[74] Camilleri M., Mckinzie S., Busciglio I., Low P., Sweetser S., Burton D., Baxter K., Ryks M., Zinsmeister A. (2008). Prospective Study of Motor, Sensory, Psychologic, and Autonomic Functions in Patients With Irritable Bowel Syndrome. Clin. Gastroenterol. Hepatol..

[75] Rodiño-Janeiro B.K., Pardo-Camacho C., Santos J., Martínez C. (2019). Mucosal RNA and protein expression as the next frontier in IBS: Abnormal function despite morphologically intact small intestinal mucosa. Am. J. Physiol. -Gastrointest. Liver Physiol..

[76] Vanheel H., Vicario M., Vanuytsel T., Van Oudenhove L., Martinez C., Keita Å.V., Pardon N., Santos J., Söderholm J.D., Tack J. (2014). Impaired duodenal mucosal integrity and low-grade inflammation in functional dyspepsia. Gut.

[77] Cirillo C., Bessissow T., Desmet A.S., Vanheel H., Tack J., Vanden Berghe P. (2015). Evidence for neuronal and structural changes in submucous ganglia of patients with functional dyspepsia. Am. J. Gastroenterol..

[78] Ducrotté P. (2012). Clinical trial: Lactobacillus plantarum 299v (DSM 9843) improves symptoms of irritable bowel syndrome. WJG.

[79] Wang J., Wang Y., Zhou H., Gu W., Wang X., Yang J. (2020). Clinical efficacy and safety of ketotifen in treating irritable bowel syndrome with diarrhea. Eur. J. Gastroenterol. Hepatol..

[80] Robles A., Perez Ingles D., Myneedu K., Deoker A., Sarosiek I., Zuckerman M.J., Schmulson M.J., Bashashati M. (2019). Mast cells are increased in the small intestinal mucosa of patients with irritable bowel syndrome: A systematic review and meta-analysis. Neurogastroenterol. Motil..

[81] Coppens D., Kips M., Stiévenard T., Mertens C., De Schepper H. (2024). Efficacy of mast cell directed therapies in irritable bowel syndrome: A systematic review. AGEB.

[82] Ng Q.X., Yau C.E., Yaow C.Y.L., Chong R.I.H., Chong N.Z.-Y., Teoh S.E., Lim Y.L., Soh A.Y.S., Ng W.K., Thumboo J. (2023). What Has Longitudinal ‘Omics’ Studies Taught Us about Irritable Bowel Syndrome? A Systematic Review. Metabolites.

[83] Kiss Z., Tél B., Farkas N., Garami A., Vincze Á., Bajor J., Sarlós P., Márta K., Erős A., Mikó A. (2018). Eosinophil Counts in the Small Intestine and Colon of Children Without Apparent Gastrointestinal Disease: A Meta-analysis. J. Pediatr. Gastroenterol. Nutr..

[84] Corinaldesi R., Stanghellini V., Cremon C., Gargano L., Cogliandro R.F., De Giorgio R., Bartesaghi G., Canovi B., Barbara G. (2009). Effect of mesalazine on mucosal immune biomarkers in irritable bowel syndrome: A randomized controlled proof-of-concept study. Aliment. Pharmacol. Ther..

[85] Barbara G., Grover M., Bercik P., Corsetti M., Ghoshal U.C., Ohman L., Rajilić-Stojanović M. (2019). Rome Foundation Working Team Report on Post-Infection Irritable Bowel Syndrome. Gastroenterology.

[86] Halliwell B., Rafter J., Jenner A. (2005). Health promotion by flavonoids, tocopherols, tocotrienols, and other phenols: Direct or indirect effects? Antioxidant or not?. Am. J. Clin. Nutr..

